# Total tumor diameter is a better indicator of multifocal papillary thyroid microcarcinoma: A propensity score matching analysis

**DOI:** 10.3389/fendo.2022.974755

**Published:** 2022-08-08

**Authors:** Ke-cheng Jiang, Bei Lin, Yu Zhang, Ling-qian Zhao, Ding-cun Luo

**Affiliations:** ^1^ The Fourth Clinical Medical College, Zhejiang Chinese Medical University, Hangzhou, China; ^2^ Department of Surgical Oncology, Affiliated Hangzhou First People’s Hospital, Zhejiang University School of Medicine, Hangzhou, China

**Keywords:** papillary thyroid microcarcinoma, total tumor diameter, propensity score matching analysis, tumor aggressiveness, multifocality

## Abstract

**Background:**

Accurate evaluation of the risk of papillary thyroid microcarcinoma (PTMC) is the key to treatment. However, the maximum diameter (MD), which is currently used in various staging systems, may not truly reflect the aggressiveness of multifocal tumors.

**Methods:**

Clinical and pathological data for 1001 patients with papillary thyroid carcinoma who underwent surgery at the Hangzhou First People’s Hospital were retrospectively analyzed. First, the relationship between total tumor diameter (TTD) and clinicopathological features in multifocal PTMC was explored. Then, patients were divided into subgroups according to the TTD. The baseline was consistent after using the propensity score matching method, and the differences between groups were compared. In addition, the effectiveness of TTD and MD in evaluating central lymph node metastasis (CLNM) was analyzed and compared.

**Results:**

TTD is associated with a range of clinicopathological features, including lymph node metastasis, extrathyroidal extension, and risk stratification. Assuming the same MD and number of foci, the invasiveness of multifocal PTMC with TTD >1 cm was significantly higher than that with TTD <1 cm, and even higher than unifocal non-PTMC. Moreover, the efficiency of TTD in predicting CLNM was also significantly higher than that of MD.

**Conclusion:**

For multifocal PTMC, TTD is a more realistic indicator of tumor biological characteristics than MD. The aggressiveness of PTMC with TTD >1 cm was significantly enhanced, and surgical treatment should be actively sought in such cases.

## Introduction

Papillary thyroid microcarcinoma (PTMC) is defined as papillary thyroid carcinoma with the largest diameter ≤1 cm. In recent years, due to its high incidence rate but relatively benign biological behavior, concerns have been raised about overdiagnosis and overtreatment of PTMC. There is a growing debate about whether active surveillance (AS) or immediate surgery are the best approaches for PTMC treatment (1, 2). The 2015 American Thyroid Association (ATA) guidelines suggest that AS can be used as an alternative to thyroidectomy for low-risk PTMC (3). However, some practitioners oppose this practice, because some PTMC cases still show aggressive characteristics, such as lymph node metastasis or extrathyroidal extension (ETE). In the latest 5th edition of the World Health Organization classification of thymoid neoplasts, PTMC is no longer considered a separate subtype, which is consistent with the management guidelines for non-PTMC (4). Therefore, the key to solving this dispute is to accurately identify the PTMC with high aggressiveness in order to make an appropriate choice between surgery and AS and provide a more personalized treatment.

Multifocality is common in PTMC, with an incidence of 26.8–36.9% according to different sources (5–7). It has been confirmed by many studies that multifocality is a risk factor affecting the aggressiveness and prognosis of thyroid carcinoma (8). However, the size of multifocal tumors in the current staging system is still described in the same way as that of unifocal tumors, focusing on the maximum diameter (MD) (9). Therefore, evaluating MD while ignoring other lesions in multifocal tumors is problematic as it may underestimate tumor grade and affect treatment. In recent years, the concept of total tumor diameter (TTD) has also been reported by many studies (10–12) and has been demonstrated to be closely related to many tumor characteristics and prognosis. Therefore, it has been suggested that TTD is closely related to tumor invasiveness.

However, previous studies were retrospective studies under natural conditions, and related confounding factors, such as MD and number of foci, were not excluded. The present study sought to evaluate the relationship between TTD and PTMC using the propensity score matching (PSM) method in order to provide an updated and more comprehensive perspective for assessing the risk of PTMC.

## Materials and methods

### Data collection

Data for pathologically confirmed PTC patients who underwent thyroid surgery in the Department of Surgical Oncology of Hangzhou First People’s Hospital between 2009 and 2020 were collected and reviewed. The exclusion criteria were as follows: 1. lack of pathological data; 2. combination with other malignant tumors; 3. second operation; 4. mixed PTC; and 5. prior non-curative surgery. The collected patient data included age, gender, MD, number of tumor foci, central lymph node metastasis (CLNM), lateral lymph node metastasis (LLNM), capsular invasion, and ETE. All of the above pathological data were independently reviewed by two experienced pathologists. In addition, all patients were assigned into low-, intermediate-, and high-risk groups based on tumor size, multifocal characteristic, lymph node involvement, and degree of ETE according to the 2015 ATA risk stratification system.

### Definition

In the present study, MD was defined as the diameter of the largest tumor focus for multifocal PTC or the primary tumor focus for unifocal PTC. Papillary thyroid carcinoma with MD >1 cm was termed non-PTMC. The multifocality was defined as two or more tumor foci within the unilateral thyroid. For multifocal PTC, TTD was defined as the total combined diameter of each tumor lesion within a lobe, and both lobes were counted separately. CLNM was defined as tumor metastasis in lymph nodes of level VI of the neck region after postoperative pathological diagnosis. LLNM was defined as tumor metastasis in lymph nodes of levels II, III, IV, and V of the neck region after postoperative pathological diagnosis. Capsular invasion was defined as the tumor involving the capsule but not breaking through after the diagnosis of postoperative pathology. ETE was defined as the tumor breaking through the thyroid capsule and invading surrounding tissues or organs. It was stratified into two levels: minimal extension (perithyroid soft tissue, sternothyroid muscles) and gross extension (subcutaneous soft tissue, larynx, trachea, esophagus, or recurrent laryngeal nerve).

### Study design and grouping

The above data were further screened and divided into three groups according to the tumor diameter and tumor multifocality as follows: Group A: multifocal PTMC with TTD ≤1 cm, Group B: multifocal PTMC with TTD >1 cm, and Group C: unifocal non-PTMC. A total of 1001 patients participated in the study, including 172 in group A, 388 in group B, and 441 in group C. The relationship between TTD and other clinicopathological features was explored first. PSM was performed based on age, sex, TTD, and number of foci between groups A and B to minimize the effect of confounders on the outcomes between these groups. In addition, PSM was also performed based on age, sex and TTD/MD between groups A and C. After reducing the impact of potential confounding factors, the clinicopathological features were compared between the groups.

Preoperative diagnosis of CLNM is a challenge for clinical decision making. The present study performed univariate logistic regression analysis on CLNM, calculated the predictive efficacy of TTD and MD, plotted receiver operating characteristic (ROC) curve, and compared the area under the curve (AUC) values.

### Surgical strategy

All patients enrolled in the study underwent radical surgery. Thyroidectomy and cervical lymph node dissection were performed simultaneously. Ipsilateral lobe and isthmus resection were performed for unilateral lesions. Total thyroidectomy was carried out for unilateral lesions requiring iodine-131 and bilateral lesions. Central lymph node dissection was performed routinely. Lateral lymph node dissection was done in patients with cervical lymph node metastasis diagnosed *via* fine-needle aspiration biopsy or preoperative imaging and confirmed by the intraoperative frozen sections.

### Statistical analysis

Categorical variables were described using frequencies and percentages. Continuous variables were expressed as the mean ± standard deviation (mean ± SD). The chi-square test or Fisher’s exact test was used to analyze categorical variables, while the t-test was applied to compare continuous variables. The “matchit” package was used to perform PSM analysis. The nearest neighbor algorithm was used as the matching method, with the ratio set to 1:1 and the caliper value set to 0.02. R software (R Core Team, Version 4.1.2, Vienna, Austria) and MedCalc software (MedCalc 19.2.1; MedCalc, Mariakerke, Belgium) were used for all data analysis in the study. Bilateral P < 0.05 served as the significance threshold.

## Results

### Comparison of baseline characteristics between groups before PSM

The clinical and pathological features were analyzed and compared between each group before PSM. First, significant differences were present in all characteristics except age (46.49 ± 12.32 vs. 47.48 ± 10.44, P = 0.748; **Table 1**) between groups A (TTD >1 cm) and B (TTD ≤1 cm). The group with TTD >1 cm had a higher proportion of males (22.09 vs. 14.95%, P = 0.039), larger MD (7.56 ± 1.60 vs. 4.54 ± 1.53, P = 0.039), and more lesions (2.45 ± 0.70 vs. 2.06 ± 0.26, P = 0.039). PTMC with TTD >1 cm also exhibited a higher level of aggressiveness, which was manifested by a higher proportion of CLNM (55.23% vs. 29.12%, P < 0.01) and LLNM (11.64% vs. 3.61%, P < 0.01). There was also a higher proportion of capsular invasion (29.65% vs. 12.37%, P < 0.01) and ETE (P < 0.01). In terms of risk stratification, patients with TTD >1 cm had a higher proportion of medium- and high-risk (P < 0.01).

**Table 1:** Comparison of baseline characteristics between multifocal PTMC with TTD >1 cm and TTD ≤1 cm before PSM.

	TTD > 1cm	TTD ≤ 1cm	P-value
Age	46.49 ± 12.32	47.48 ± 10.44	
<55	129 (75%)	286 (73.71%)	0.748
≥55	43 (25%)	102 (26.29%)	
Gender			
Female	134 (77.91%)	330 (85.05%)	0.039
Male	38 (22.09%)	58 (14.95%)	
Maximum diameter	7.56 ± 1.60	4.54 ± 1.53	
1	0 (0%)	4 (1%)	<0.01
2	0 (0%)	27 (6.96%)	
3	1 (0.58%)	73 (18.81%)	
4	4 (2.33%)	91 (23.45%)	
5	13 (7.56%)	94 (24.23%)	
6	27 (15.70%)	57 (14.69%)	
7	35 (20.35%)	31 (7.99%)	
8	43 (25.00%)	9 (2.32%)	
9	25 (14.53%)	2 (0.52%)	
10	24 (13.95%)	0 (0%)	
Number of tumor foci	2.45 ± 0.70	2.06 ± 0.26	
2	111 (64.53%)	386 (93.81%)	<0.01
3	49 (28.49%)	23 (5.93%)	
4	9 (5.23%)	1 (0.26%)	
5	2 (1.16%)	0 (0%)	
6	1 (0.58%)	0 (0%)	
CLNM			
Negative	77 (44.77%)	275 (70.88%)	<0.01
Positive	95 (55.23%)	113 (29.12%)	
LLNM			
Negative	152 (88.37%)	374 (96.39%)	<0.01
Positive	20 (11.64%)	14 (3.61%)	
Capsular invasion			<0.01
Negative	121 (70.35%)	340 (87.63%)	
Positive	51 (29.65%)	48 (12.37%)	
Extrathyroidal extension			<0.01
Intrathyroidal	129 (75.00%)	354 (91.24%)	
Minimal extension	39 (22.67%)	34 (8.76%)	
Gross extension	4 (2.33%)	0 (0%)	
Risk stratification			<0.01
Low	117 (68.02%)	359 (92.53%)	
Medium	51 (29.65%)	29 (7.47%)	
High	4 (2.33%)	0 (0%)	

Groups A and C were then compared. The baseline comparison between the two groups before PSM is shown in **Table 2**. There was no significant difference in age and sex between the two groups, but the baseline TTD/MD comparison was inconsistent (13.19 ± 2.35 vs. 14.14 ± 2.78, P < 0.01). No difference in CLNM, capsular invasion, and risk stratification was found between the two groups, while the non-PTMC group showed a higher proportion in LLNM (11.64% vs. 22.90%, P < 0.01). In terms of ETE, the multifocal PTMC group showed a higher proportion of medium- and high-risk.

**Table 2:** Comparison of baseline characteristics between multifocal PTMC and unifocal non-PTMC before PSM.

	Multifocal PTMC	Unifocal non-PTMC	P-value
Age	46.49 ± 12.32	46.27 ± 13.00	
<55	129 (75%)	311 (70.52%)	0.268
≥55	43 (25%)	130 (29.48%)	
Gender			
Male	134 (77.91%)	334 (78.00%)	0.979
Female	38 (22.09%)	97 (22.00%)	
Maximum diameter	13.19 ± 2.35	14.14 ± 2.78	
11	50 (29.07%)	65 (14.74%)	<0.01
12	45 (26.16%)	107 (24.26%)	
13	17 (9.88%)	57 (12.93%)	
14	17 (9.88%)	38 (8.62%)	
15	10 (5.81%)	77 (17.46%)	
16	10 (5.81%)	7 (1.59%)	
17	10 (5.81%)	13 (2.95%)	
18	9 (5.23%)	31 (7.03%)	
19	2 (1.16%)	4 (0.91%)	
20	2 (1.16%)	42(9.52%)	
CLNM			
Negative	77 (44.77%)	212 (48.07%)	0.461
Positive	95 (55.23%)	229 (51.93%)	
LLNM			
Negative	152 (88.37%)	340 (77.10%)	<0.01
Positive	20 (11.64%)	101 (22.90%)	
Capsular invasion			
Negative	121 (70.35%)	330 (74.83%)	0.258
Positive	51 (29.65%)	111 (25.17%)	
Extrathyroidal extension			
Intrathyroidal	129 (75.00%)	380 (86.17%)	<0.01
Minimal extension	39 (22.67%)	45 (10.20%)	
Gross extension	4 (2.33%)	16 (3.63%)	
Risk stratification			
Low	117 (68.02%)	318 (72.11%)	0.222
Medium	51 (29.65%)	205 (23.81%)	
High	4 (2.33%)	18 (4.08%)	

### Relationship between TTD and clinicopathological features in PTMC

**Figure 1:**
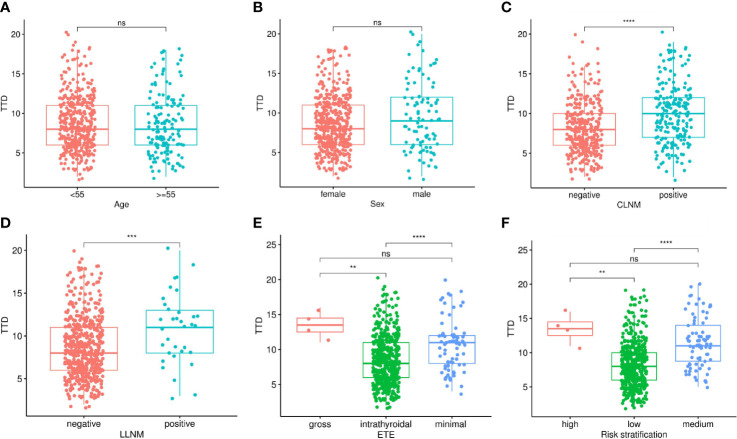
Relationship between TTD and clinicopathological features in multifocal PTMC, including Age **(A)**, Sex **(B)**, CLNM **(C)**, LLNM **(D)**, ETE **(E)** and Risk stratification **(F)**. TTD, total tumor diameter; PTMC, papillary thyroid microcarcinoma; CLNM, central lymph node metastasis; LLNM, lateral lymph node metastasis; ETE, extrathyroidal extension. *P < 0.05; **P < 0.01; ***P < 0.001; ****P < 0.0001; ns, not significant.

The relationship between TTD and various clinicopathological features was explored in 560 PTMC patients included in the study. The results showed that there is no difference in TTD between different ages and genders, which means that there is no correlation between them (**Figure 1**). For lymph node metastasis, both CLNM and LLNM were significantly correlated with TTD. The TTD of tumors with metastasis was higher than that of tumors without metastasis (**Figure 1**). There were significant differences between intrathyroidal and extensional tumors in terms of ETE, but no differences between minimal and gross extension. The risk stratification was similar (**Figure 1**). The TTD of the medium- and high-risk groups was significantly higher than that of the low-risk group, but there was no difference between the medium- and high-risk groups (**Figure 1**).

### Comparison of clinicopathological features between multifocal PTMC with TTD >1 cm and TTD <1 cm

A total of 124 patients were included in the analysis after 1:1 PSM was carried out. There was no significant difference in age, sex, MD, and number of lesions between the two groups (**Table 3**). Interestingly, there were also significant differences between the two groups in terms of other aspects, except LLNM. CLNM (61.29% vs. 41.94%, P < 0.01), capsular invasion (P < 0.01), and ETE (P < 0.01) were greater in the TTD >1 cm group. However, the vast majority of ETE cases could be classified as those with minimal extension, while the proportion of gross extension was very low. In terms of risk stratification, the TTD >1 cm group included more medium-risk patients, while the number of high-risk patients was lower in both groups.

**Table 3:** Comparison of clinicopathological features between multifocal PTMC with TTD >1 cm and TTD <1 cm after PSM.

	TTD > 1cm	TTD ≤ 1cm	P-value
Age	46.37 ± 11.61	45.08 ± 9.33	
<55	48 (77.42%)	53 (85.48%)	0.248
≥55	14 (22.58%)	9(14.52%)	
Gender			
Male	53 (85.48%)	54 (87.10%)	0.794
Female	9 (14.52%)	8 (12.90%)	
Maximum diameter	6.44 ± 0.99	6.73 ± 0.98	
3	1 (1.61%)	0 (0%)	0.489
4	2 (3.23%)	1(1.61%)	
5	4 (6.45%)	5 (8.06%)	
6	22 (35.48%)	17 (27.42%)	
7	29 (46.77%)	28 (45.16%)	
8	3 (4.84%)	9 (14.52%)	
9	1 (1.61%)	2 (3.23%)	
Number of tumor foci	2.29 ± 0.58	2.15 ± 0.40	
2	111 (64.53%)	386 (93.81%)	0.350
3	49 (28.49%)	23 (5.93%)	
4	9 (5.23%)	1 (0.26%)	
5	2 (1.16%)	0 (0%)	
CLNM			
Negative	24 (38.71%)	36 (58.06%)	0.031
Positive	38 (61.29%)	26 (41.94%)	
LLNM			
Negative	58 93.55%)	58 (93.55%)	1
Positive	4 (6.45%)	4 (6.45%)	
Capsular invasion			
Negative	44 (70.97%)	56 (90.32%)	<0.01
Positive	18 (29.03%)	6 (12.37%)	
Extrathyroidal extension			
Intrathyroidal	46 (74.19%)	57 (91.94%)	0.028
Minimal extension	15 (24.19%)	5 (8.06%)	
Gross extension	1 (1.61%)	0 (0%)	
Risk stratification			
Low	117 (68.02%)	359 (92.53%)	0.041
Medium	51 (29.65%)	29 (7.47%)	
High	4 (2.33%)	0 (0%)	

### Comparison of clinicopathological features between multifocal PTMC and unifocal non-PTMC

Groups A and C were compared in order to further explore the effect of TTD on invasiveness. A total of 224 patients were included after 1:1 PSM was performed. The interference of confounding factors was eliminated and the baseline was consistent (**Table 4**). The results showed that there was no significant difference in CLNM between the two groups, while the non-PTMC group had a higher proportion of LLNM. Different from the general belief that PTMC exhibits a benign biological behavior and similar to the TTD/MD results, the multifocal PTMC group demonstrated a greater level of capsular invasion (P < 0.01) and more cases of ETE (P < 0.01). There were also differences in risk stratification. Specifically, there were more medium-risk patients in the multifocal PTMC group and more high-risk patients in the unifocal non-PTMC group.

**Table 4:** Comparison of clinicopathological features between multifocal PTMC and unifocal non-PTMC after PSM.

	Multifocal PTMC	Unifocal non-PTMC	P-value
Age	46.51 ± 12.17	45.76 ± 11.78	
<55	125 (75.30%)	125 (75.30%)	1
≥55	41(24.70%)	41 (24.70%)	
Gender			
Female	134 (80.72%)	136 (81.93%)	0.979
Male	32 (19.28%)	30 (18.07%)	
Maximum diameter	13.08 ± 2.32	13.08 ± 2.32	
11	50 (30.12%)	50 (30.12%)	1
12	45(27.11%)	45(27.11%)	
13	17 (10.24%)	17 (10.24%)	
14	17 (10.24%)	17 (10.24%)	
15	10 (6.02%)	10 (6.02%)	
16	5 (3.01%)	5 (3.01%)	
17	9 (5.42%)	9 (5.42%)	
18	9 (5.42%)	9 (5.42%)	
19	2 (1.20%)	2 (1.20%)	
20	2 (1.20%)	2 (1.20%)	
CLNM			
Negative	75 (45.18%)	81 (48.80%)	0.509
Positive	91(54.82%)	85 (51.20%)	
LLNM			
Negative	147 (88.55%)	132 (79.52%)	0.025
Positive	19(11.45%)	34 (20.48%)	
Capsular invasion			
Negative	118 (71.08%)	145 (87.35%)	<0.01
Positive	48 (28.92%)	21 (12.65%)	
Extrathyroidal extension			
Intrathyroidal	126 (75.90%)	156 (93.95%)	<0.01
Minimal extension	37 (22.29%)	6 (3.61%)	
Gross extension	3 (1.81%)	4 (2.41%)	
Risk stratification			
Low	115 (69.28%)	133 (80.12%)	0.046
Medium	48(28.92%)	29 (17.47%)	
High	3 (1.81%)	18 (2.41%)	

### Comparison of prediction efficiency of TTD and MD for CLNM

**Figure 2:**
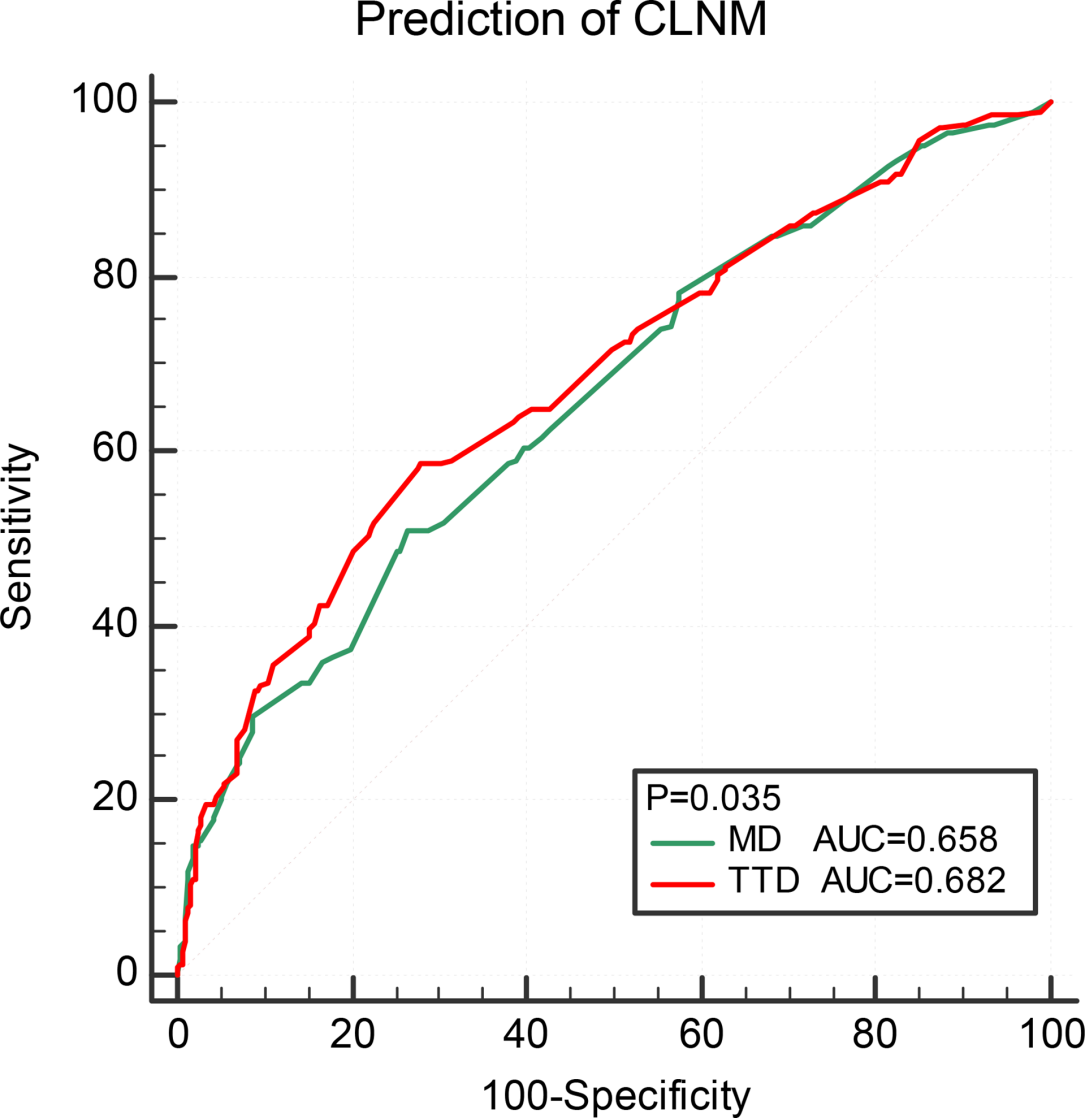
Comparison of prediction efficiency of TTD and MD for CLNM. TTD, total tumor diameter; CLNM, central lymph node metastasis; ETE, extrathyroidal extension.

Multivariate logistic regression analysis was performed on MD and TTD, combining age, sex, and ETE (**Table 5**). The respective calculated AUC values of 0.658 and 0.682 were statistically significantly different. This suggests that TTD may be a better predictor of CLNM for multifocal PTMC (**Figure 2**).

**Table 5:** Logistic regression analysis of CLNM-related risk factors.

Variate	Odds ratio	95% CI	P-value	Variate	Odds ratio	95% CI	P-value
MD	1.24	1.134–1.356	<0.01	TTD	1.17	1.106–1.231	<0.01
Age (vs <55)	0.60	0.393–0.923	0.02	Age (vs <55)	0.60	0.391–0.926	0.02
Sex (vs male)	0.46	0.292–0.739	0.01	Sex (vs male)	0.49	0.302–0.780	0.01
ETE (vs intrathyroidal)				ETE (vs intrathyroidal)			
Minimal extension	1.26	0.742–2.138	0.393	minimal extension	1.20	0.702–2.055	0.393
Gross extension	3.61	0.358–36.462	0.276	gross extension	3.61	0.245–27.479	0.276

## Discussion

The significance of multifocality, as well as total diameter, has been reported in other cancers. For example, in breast cancer it is associated with a higher rate of metastasis and worse prognosis (13, 14), and in renal cancer it is associated with adverse biological characteristics (15). The significance of TTD in thyroid cancer has also been reported in previous studies. By reviewing 275 patients with thyroid cancer, Zhao et al. (10) have found that the risk of CLNM was significantly increased in patients with multifocal PTMC with TTD >1 cm and have recommended that central neck dissection should be performed routinely. Tam et al. (16) have grouped 912 patients according to TTD and found that PTMC with TTD >1 cm confers a similar risk of aggressive histopathological behavior as uniform non-PMTC with MD >1 cm. Feng et al. (12) have analyzed 442 patients and found that TTD was associated with lymph node metastasis and ETE and suggested that TTD can better assess tumor aggressiveness. However, the above studies did not mention the results after adjusting for confounding factors, such as age, gender, and MD, which are related to the characteristics of tumors. The present study used a PSM method to explore the relationship between TTD and clinicopathological features based on about 1000 thyroid cancer patients in our center.

CLNM is common among PTMC patients, but due to its specific anatomical location, the current imaging methods have insufficient diagnostic ability (17). In other words, clinical lymph nodes negative(cN0) is not reliable. There is still controversy about whether cN0 PTC patients are eligible for prophylactic central neck dissection (pCND) (18, 19). Therefore, the identification of high-risk factors for CLNM is helpful to guide clinical decision making. TTD in the present study was significantly correlated with CLNM. After PSM, 61.29% of patients in group A had CLNM, which was significantly higher than the level in group B and no different compared to group C. In addition, the efficiency of TTD in predicting CLNM is significantly higher than that of MD in PTMC. This suggests that TTD may be a better predictor of CLNM for multifocal PTMC. Moreover, for PTMC with TTD >1 cm, a more positive attitude towards pCND should be adopted. The general incidence of PTMC for LLNM was low at ~6.45%. Although TTD was correlated with it, there was no difference in metastasis rate between groups A and B. There are several possible reasons for this result. First of all, the capsular invasion and ETE are generally lower in PTMC. Tumor cells metastasize station by station along lymphatic drainage and follow the rule of progressive spread through the cervical lymphatic network. Second, there were few patients with LLNM in this study, making it difficult to demonstrate statistical difference. If more samples can be included in the future, the conclusion will be more convincing. Third, our center is located in economically developed areas, and most of the cases are early tumors found *via* physical examination, resulting in a certain selection bias. Multi-center studies will be carried out in the future. Therefore, preoperative LLNM diagnosis needs to be executed with care.

ETE is an important factor affecting the invasiveness of thyroid cancer and gross ETE is usually considered to be an indication for near-total or total thyroidectomy (20). Accurate preoperative prediction of ETE can help doctors to determine appropriate surgical strategies to reduce the risk of reoperation. TTD was correlated with ETE among the patients in the present study, which was consistent with the results of previous studies. In addition, with the same MD and number of foci values, the occurrence of ETE in PTMC with TTD >1 cm was significantly higher than that with TTD <1 cm. The results suggest that PTMC patients with TTD >1 cm need a more thorough surgical and radioactive iodine therapy. However, it should be noted that most of the cases had a minimal extension, while PTMC appearance with gross extension was very rare in the present cohort, which is significantly less than the values for non-PTMC. At present, there is controversy about minimal ETE (21, 22). Some studies have shown that minimal ETE is associated with adverse biological behavior, but not prognosis. However, this conclusion may still need more studies to verify.

The relationship between TTD and risk stratification is also discussed. ATA risk stratification is one of the most widely used risk stratification systems for assessing an individual patient’s risk of persistent or recurrent disease (23). It helps doctors to determine the necessity of postoperative radioactive iodine treatment, degree of TSH inhibition, and follow-up frequency (24). In this study, the ATA risk strategy was used to directly evaluate the impact on diagnosis The recurrence rate of low, medium and high risk was 1–5%, 5–20%, and >20%, respectively (25). It is possible that this can reveal the impact of TTD on prognosis to a certain extent. Although tumor size was not included in the risk stratification analysis, the present study found that there was a correlation between TTD and stratification. PTMC with TTD >1 cm had a greater proportion of medium- and high-risk cases than PTMC with TTD <1 cm. Therefore, AS is not suitable as a treatment for PTMC patients with TTD >1 cm, and immediate surgery is a better choice.

This research study has some limitations. First, the follow-up data are lacking in this study due to the large time span. Second, this was a single center study. Data from more centers should be included in the future. Third, lateral neck lymph node dissection was not performed on all patients. Therefore, occult LLNM may have been present.

## Conclusion

In conclusion, the present study provides new evidence for the relationship between TTD and PTMC. For multifocal PTMC, TTD can better reflect the tumor biological characteristics and aggressiveness than MD. The aggressiveness was significantly increased for PTMC with TTD >1 cm and a more active treatment should be provided for such patients.

## Data availability statement

The original contributions presented in the study are included in the article/supplementary material. Further inquiries can be directed to the corresponding author.

## Ethics statement

Written informed consent was obtained from the individual(s) for the publication of any potentially identifiable images or data included in this article. 

## Author contributions

K-cJ, conception and design, provision of study materials or patients, data analysis and interpretation, and manuscript writing. BL, conception and design, provision of study materials or patients, data analysis and interpretation, and manuscript writing. L-qZ, provision of study materials or patients and manuscript writing. YZ, provision of study materials or patients and manuscript writing. D-cL, conception and design, administrative support, provision of study materials or patients, and manuscript writing. All authors contributed to the article and approved the submitted version.

## Funding

This work was supported by the Science and Technology Department of Zhejiang Province (Grant numbers GF22H165705), the Medical and Health Technology Project of Hangzhou (Grant number Z20210025), and the Zhejiang Provincial Medical and Health Technology Project (Grant number 2022KY939).

## Conflict of interest

The authors declare that the research was conducted in the absence of any commercial or financial relationships that could be construed as a potential conflict of interest.

## Publisher’s note

All claims expressed in this article are solely those of the authors and do not necessarily represent those of their affiliated organizations, or those of the publisher, the editors and the reviewers. Any product that may be evaluated in this article, or claim that may be made by its manufacturer, is not guaranteed or endorsed by the publisher.

## References

[1] Brito JP Hay ID. Management of papillary thyroid microcarcinoma. Endocrinol Metab Clinics North America (2019) 48(1):199–213. doi: 10.1016/j.ecl.2018.10.006 30717902

[2] Cernea CR Matos LL Eugenio C Ferreira GM Cerqueira YS Leite AKN. Active surveillance of thyroid microcarcinomas: A critical view. Curr Oncol Rep (2022) 24(1):69–76. doi: 10.1007/s11912-021-01177-w 35061193

[3] Haugen BR Alexander EK Bible KC Doherty GM Mandel SJ Nikiforov YE. 2015 American Thyroid association management guidelines for adult patients with thyroid nodules and differentiated thyroid cancer: The American thyroid association guidelines task force on thyroid nodules and differentiated thyroid cancer. Thyroid (2016) 26(1):1–133. doi: 10.1089/thy.2015.0020 26462967 PMC4739132

[4] Baloch ZW Asa SL Barletta JA Ghossein RA Juhlin CC Jung CK. Overview of the 2022 who classification of thyroid neoplasms. Endocr Pathol (2022) 33(1):27–63. doi: 10.1007/s12022-022-09707-3 35288841

[5] Pyo JS Sohn JH Kang G. Detection of tumor multifocality is important for prediction of tumor recurrence in papillary thyroid microcarcinoma: A retrospective study and meta-analysis. J Pathol Transl Med (2016) 50(4):278–86. doi: 10.4132/jptm.2016.03.29 PMC4963970 27271109

[6] Luo Y Zhao Y Chen K Shen J Shi J Lu S. Clinical analysis of cervical lymph node metastasis risk factors in patients with papillary thyroid microcarcinoma. J Endocrinol Invest (2019) 42(2):227–36. doi: 10.1007/s40618-018-0908-y PMC6394766 29876836

[7] Song J Yan T Qiu W Fan Y Yang Z. Clinical analysis of risk factors for cervical lymph node metastasis in papillary thyroid microcarcinoma: A retrospective study of 3686 patients. Cancer Manag Res (2020) 12:2523–30. doi: 10.2147/CMAR.S250163 PMC7153998 32308489

[8] Feng JW Qu Z Qin AC Pan H Ye J Jiang Y. Significance of multifocality in papillary thyroid carcinoma. Eur J Surg Oncol (2020) 46(10 Pt A):1820–8. doi: 10.1016/j.ejso.2020.06.015 32732090

[9] Perrier ND Brierley JD Tuttle RM. Differentiated and anaplastic thyroid carcinoma: Major changes in the American joint committee on cancer eighth edition cancer staging manual. CA Cancer J Clin (2018) 68(1):55–63. doi: 10.3322/caac.21439 29092098 PMC5766386

[10] Zhao Q Ming J Liu C Shi L Xu X Nie X. Multifocality and total tumor diameter predict central neck lymph node metastases in papillary thyroid microcarcinoma. Ann Surg Oncol (2013) 20(3):746–52. doi: 10.1245/s10434-012-2654-2 22972508

[11] Liu C Wang S Zeng W Guo Y Liu Z Huang T. Total tumour diameter is superior to unifocal diameter as a predictor of papillary thyroid microcarcinoma prognosis. Sci Rep (2017) 7(1):1846. doi: 10.1038/s41598-017-02165-6 28500312 PMC5431972

[12] Feng JW Pan H Wang L Ye J Jiang Y Qu Z. Total tumor diameter: The neglected value in papillary thyroid microcarcinoma. J Endocrinol Invest (2020) 43(5):601–13. doi: 10.1007/s40618-019-01147-x 31749082

[13] Karakas Y Dizdar O Aksoy S Hayran M Altundag K. The effect of total size of lesions in Multifocal/Multicentric breast cancer on survival. Clin Breast Cancer (2018) 18(4):320–7. doi: 10.1016/j.clbc.2017.11.002 29183716

[14] Cabioglu N Ozmen V Kaya H Tuzlali S Igci A Muslumanoglu M. Increased lymph node positivity in multifocal and multicentric breast cancer. J Am Coll Surg (2009) 208(1):67–74. doi: 10.1016/j.jamcollsurg.2008.09.001 19228505

[15] Siracusano S Novara G Antonelli A Artibani W Bertini R Carini M. Prognostic role of tumour multifocality in renal cell carcinoma. BJU Int (2012) 110(11b):E443–E8. doi: 10.1111/j.1464-410X.2012.11121.x 22502873

[16] Tam AA Ozdemir D Ogmen BE Faki S Dumlu EG Yazgan AK. Should multifocal papillary thyroid carcinomas classified as T1a with a tumor diameter sum of 1 to 2 centimeters be reclassified as T1b? Endocr Pract (2017) 23(5):526–35. doi: 10.4158/EP161488.OR 28156153

[17] Al Afif A Williams BA Rigby MH Bullock MJ Taylor SM Trites J. Multifocal papillary thyroid cancer increases the risk of central lymph node metastasis. Thyroid (2015) 25(9):1008–12. doi: 10.1089/thy.2015.0130 26161997

[18] Sippel RS Robbins SE Poehls JL Pitt SC Chen H Leverson G. A randomized controlled clinical trial: No clear benefit to prophylactic central neck dissection in patients with clinically node negative papillary thyroid cancer. Ann Surg (2020) 272(3):496–503. doi: 10.1097/SLA.0000000000004345 33759836 PMC8496479

[19] McHenry CR Stulberg JJ. Prophylactic central compartment neck dissection for papillary thyroid cancer. Surg Clin North Am (2014) 94(3):529–40. doi: 10.1016/j.suc.2014.02.003 24857575

[20] Youngwirth LM Adam MA Scheri RP Roman SA Sosa JA. Extrathyroidal extension is associated with compromised survival in patients with thyroid cancer. Thyroid (2017) 27(5):626–31. doi: 10.1089/thy.2016.0132 27597378

[21] Moon HJ Kim EK Chung WY Yoon JH Kwak JY. Minimal extrathyroidal extension in patients with papillary thyroid microcarcinoma: Is it a real prognostic factor? Ann Surg Oncol (2011) 18(7):1916–23. doi: 10.1245/s10434-011-1556-z 21267788

[22] Seifert R Schafers MA Heitplatz B Kerschke L Riemann B Noto B. Minimal extrathyroid extension in papillary micro carcinoma of the thyroid is an independent risk factor for relapse through lymph node and distant metastases. J Nucl Med (2021) 62(12):1702–09. doi: 10.2967/jnumed.121.261898 PMC8612207 33771902

[23] Stefanova DI Bose A Ullmann TM Limberg JN Finnerty BM Zarnegar R. Does the ata risk stratification apply to patients with papillary thyroid microcarcinoma? World J Surg (2020) 44(2):452–60. doi: 10.1007/s00268-019-05215-4 31605172

[24] van Velsen EFS Stegenga MT van Kemenade FJ Kam BLR van Ginhoven TM Visser WE. Evaluating the 2015 American thyroid association risk stratification system in high-risk papillary and follicular thyroid cancer patients. Thyroid (2019) 29(8):1073–9. doi: 10.1089/thy.2019.0053 31140385

[25] Choudhury S Agrawal A Pantvaidya G Shah S Purandare N Puranik A. Assessment of the impact of 2015 American thyroid association guidelines in management of differentiated thyroid cancer patients. Eur J Nucl Med Mol Imaging (2020) 47(3):547–53. doi: 10.1007/s00259-019-04582-3 31707429

